# Gene regulation associated with sexual development and female fertility in different isolates of *Trichoderma reesei*

**DOI:** 10.1186/s40694-018-0055-4

**Published:** 2018-05-15

**Authors:** Christoph Dattenböck, Doris Tisch, Andre Schuster, Alberto Alonso Monroy, Wolfgang Hinterdobler, Monika Schmoll

**Affiliations:** 1 Center for Health and Bioresources, AIT Austrian Institute of Technology GmbH, Konrad Lorenz Straße 24, 3430 Tulln, Austria; 2 Institute of Chemical Engineering, Research Area Molecular Biotechnology, TU Wien, 1060 Vienna, Austria

**Keywords:** *Trichoderma reesei*, *Hypocrea jecorina*, Sexual development, Female fertility, Secondary metabolism, Mating type

## Abstract

**Background:**

*Trichoderma reesei* is one of the most frequently used filamentous fungi in industry for production of homologous and heterologous proteins. The ability to use sexual crossing in this fungus was discovered several years ago and opens up new perspectives for industrial strain improvement and investigation of gene regulation.

**Results:**

Here we investigated the female sterile strain QM6a in comparison to the fertile isolate CBS999.97 and backcrossed derivatives of QM6a, which have regained fertility (FF1 and FF2 strains) in both mating types under conditions of sexual development. We found considerable differences in gene regulation between strains with the CBS999.97 genetic background and the QM6a background. Regulation patterns of QM6a largely clustered with the backcrossed FF1 and FF2 strains. Differential regulation between QM6a and FF1/FF2 as well as clustering of QM6a patterns with those of CBS999.97 strains was also observed. Consistent mating type dependent regulation was limited to mating type genes and those involved in pheromone response, but included also *nta1* encoding a putative N-terminal amidase previously not associated with development. Comparison of female sterile QM6a with female fertile strains showed differential expression in genes encoding several transcription factors, metabolic genes and genes involved in secondary metabolism.

**Conclusions:**

Evaluation of the functions of genes specifically regulated under conditions of sexual development and of genes with highest levels of transcripts under these conditions indicated a relevance of secondary metabolism for sexual development in *T. reesei*. Among others, the biosynthetic genes of the recently characterized SOR cluster are in this gene group. However, these genes are not essential for sexual development, but rather have a function in protection and defence against competitors during reproduction.

**Electronic supplementary material:**

The online version of this article (10.1186/s40694-018-0055-4) contains supplementary material, which is available to authorized users.

## Background

The parental strain of *Trichoderma reesei* (syn. *Hypocrea jecorina*) strains that dominated research and industry, QM6a, has been isolated during WWII in the tropics [1–3]. For decades it was considered asexual, which was a drawback for genetics research and industrial applications [4]. In 2009, sexual development of *T. reesei* under laboratory conditions was achieved [5, 6], albeit at the same time, female sterility of QM6a was discovered. Later on, a mutation in the gene encoding the WD-40 protein IDC1/HAM-5 [7, 8] was identified to cause female sterility in QM6a [9, 10].

Sexual development is dependent on the presence of a functional pheromone system in fungi [11] as well as on precisely defined environmental conditions [12, 13]. In *T. reesei*, in contrast to many other fungi, sexual development is initiated upon growth on complex media such as malt extract agar (MEA) or potato dextrose agar (PDA) and occurs preferentially in light [14]. *T. reesei* requires one of the two mating type associated pheromone precursor—pheromone receptor pairs (*hpp1*–*hpr1* or *ppg1*–*hpr2*) to be functional in order to undergo mating successfully [15]. Interestingly, *T. reesei* has no conventional a-type peptide pheromone precursor, but employs a novel type of pheromones, with HPP1 as the first representative of h-type pheromones [16].

The blue light photoreceptor ENV1 is crucial for light dependent balancing of regulation of the pheromone system. Its abolishment causes female sterility in light due to deregulation of the expression of pheromone receptor and precursor genes, predominantly in the mating type MAT1-2 [17]. This female sterility is conditional and can be overcome by application of an altered light regime [18]. In contrast to ENV1, the BLR1-BLR2 photoreceptor is not essential for sexual development as mutation of the genes causes only minor modulations in its efficiency as well as some morphological alterations in the fruiting body [17, 19].

Besides the light signaling pathway also protein kinase A and adenylate cyclase, the major components of the cAMP pathway of *T. reesei,* influence efficiency of sexual development [20]. For the heterotrimeric G-protein pathway, functions in sexual development are known for the G-protein beta and gamma subunits GNB1 and GNG1 [21].

Chemical communication via the secretion of secondary metabolites was shown to be important for sexual development in *T. reesei*. The pattern of secondary metabolites secreted into the medium changes if a compatible mating partner is sensed. Thereby, VEL1 was found to be crucial for triggering recognition associated signaling [22]. Secretion of secondary metabolites is regulated by light in *T. reesei* and a connection to carbon catabolite repression has been shown [23]. Moreover, among the genes regulated in a cellulase induction specific manner, several secondary metabolism associated genes were found, including the polyketide synthase *pks4* [24], which is responsible for the green coloration of spores of *T. reesei* among other functions [25].

The genome of the natural isolate CBS999.97 [6, 26] was published recently [10] and showed a particularly low occurrence of non-synonymous SNPs within genesets enriched in functions of metabolism, signal transduction and stress response, while genes comprising a high number of SNPs are involved in secondary metabolism or photoperception [10]. Comparative analysis of CBS999.97, QM6a and FF1/FF2, female fertile strains backcrossed from CBS999.97 to gain the QM6a phenotype revealed different carbon utilization characteristics between the two strain backgrounds. Additionally, secondary metabolite profiles were different between CBS999.97 and QM6a and regulatory differences associated with female fertility and female sterility were detected, which include regulation of CAZyme and transporter encoding genes [10].

In this study we investigated the transcriptome of QM6a representing strains applied in research and industry as well as those of the female fertile isolate CBS999.97 and backcrossed strains (FF1, FF2) under conditions of sexual development. Besides differential gene regulation between different mating types and strain backgrounds, we also found altered regulation between female fertile strains and QM6a. Moreover, a relevance of secondary metabolism for sexual development in *T. reesei* became obvious.

## Results

### Gene expression patterns in strains with QM6a background versus CBS999.97

**Fig. 1:**
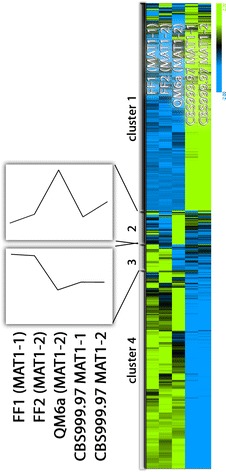
Hierarchical clustering of gene expression patterns. Regulation patterns under conditions of sexual development (22 °C, malt extract agar 2% w/v, light–dark cycles) of strains with different genetic background. Average expression patterns for clusters 2 and 3 are shown separately

We performed transcriptome analysis under conditions facilitating sexual development and enable the associated chemical communication using the wild-isolate CBS999.97 in both mating types [6], the female sterile strain QM6a and female fertile derivatives of QM6a in both mating types (FF1 and FF2), which were prepared by repeated backcrossing [10, 22]. Hierarchical cluster analysis of gene expression revealed 4 clusters (Fig. 1). This analysis clearly showed that under conditions of sexual development, QM6a is more similar to FF1 and FF2 than to CBS999.97 and hence confirms that the backcrossing procedure largely restored the QM6a phenotype. A similar result was also achieved for conditions of cellulase gene expression [10].

Of the identified 4 clusters, clusters 2 and 3 show the most interesting patterns (Fig. 1). Cluster 2 comprises genes upregulated in QM6a compared to FF1/FF2 as well as CBS999.97. Functional category analysis of these genes showed numerous metabolic genes in this cluster, but significant enrichment (p-values below 5E − 03) was only found for functions in RNA processing, sesquiterpenes metabolism, cellular import and defense. In cluster 3 there are genes for which QM6a clusters with CBS999.97 rather than FF1/FF2. This gene set was enriched for functions in detoxification as well as C-1 compound metabolism. Interestingly, this gene set also contained an unusually high proportion of unclassified proteins suggesting that yet unknown functions may be shared by QM6a and CBS999.97.

Hierarchical clustering of the subset of genes annotated as involved in sexual development [3] showed a comparable distribution, and a clear similarity between QM6a and FF1/FF2 (data not shown).

### Gene regulation in female fertile strains compared to female sterile QM6a

We were interested whether the difference between female fertility and female sterility is reflected in differential gene expression between crossings of QM6a and those of female fertile strains. Therefore we first compared gene regulation in FF2 strains versus QM6a. We found 210 genes to be up-regulated and 170 genes to be downregulated in FF2 strains compared to QM6a (Additional file 1). In order to get a more robust evaluation of potential alterations in QM6a, we checked how many of these genes are consistently regulated in CBS999.97 MAT1-2. Only 93 of the upregulated genes are also upregulated in CBS999.97 MAT1-2 and 74 of the down regulated genes show the same regulation in this strain (Additional file 1). The gene set of upregulated genes in female fertile MAT1-2 strains was significantly enriched in functions of drug/toxin transport (*p* value 1.39e−04), type I protein secretion (p-value 1.20e−03), disease, virulence and defense (p-value 3.28e−03) and detoxification by export (p-value 1.80e−03).

Interestingly, also the gene encoding SOR4/TR_43701, which was recently shown to influence production of sorbicillin derivatives in *T. reesei* [23], was among these genes. Its deletion does not impact sexual development of *T. reesei* (A. Monroy, unpublished results). Further up-regulated genes include a *ccg*-*13* homologue potentially involved in asexual development, three CAZyme encoding genes, 6 transcription factor genes and several predicted transporter genes (Additional file 1).

Genes down-regulated in female fertile MAT1-2 compared to QM6a were significantly enriched in functions in metabolism (p-value 5.89e−04), particularly secondary metabolism (p-value 2.46e−05). However, also functions in disease, virulence and defense were enriched, indicating a consistent shift in regulation of genes within similar functional groups between female fertile MAT1-2 strains and QM6a. This gene set comprises moreover three transcription factors, several transporters as well as the polyketide synthase gene *pks6g* and the terpenoid synthase encoding *tps7*. Interestingly, also *lae1,* which encodes a putative methyltransferase and impacts secondary metabolism in several fungi [27–29] is up to three-fold downregulated compared to QM6a (Additional file 1).

### Few genes only are consistently differentially regulated in different mating types

Differences in gene expression between different mating types have been reported previously, also in *T. reesei* [10]. Therefore we were interested whether such differences are detectable in *T. reesei* under conditions of sexual development and if they are consistent in different strain backgrounds (CBS999.97 vs. FF1/FF2 which have the QM6a genetic background).

In CBS999.97 we found 18 genes to be differentially regulated between MAT1-1 and MAT1-2 (at least twofold, p-value 0.01), 12 were downregulated and 6 were upregulated in MAT1-1 (Additional file 2). For FF1/FF2 we detected differential regulation for 39 genes, with 13 genes upregulated in FF1 and 26 genes downregulated. Of those genes, only 6 were consistently regulated in CBS999.97 and FF1/FF2 and can hence be considered consistently mating type regulated in *T. reesei*. These genes comprise the two peptide pheromone receptor genes *hpr1* and *hpr2*, as well as the mating type genes *mat1*-*2*-*1*, *mat1*-*1*-*1* and *mat1*-*1*-*3*. Thereby, *hpr1* is up-regulated in MAT1-1 and *hpr2* is upregulated in MAT1-2, as would be expected due to the associate mating types [15]. The genes of the mating type locus can be considered to be regulated above background, as they are not present in the opposite mating types and show up-regulation in their cognate mating types (*mat1*-*2*-*1* in MAT1-2; *mat1*-*1*-*1* and *mat1*-*1*-*3* in MAT1-1). In contrast, *mat1*-*1*-*2* was not found to be regulated and only very low transcript levels we observed, indicating that this gene may be relevant for a different stage of sexual development than investigated here.

**Fig. 2:**
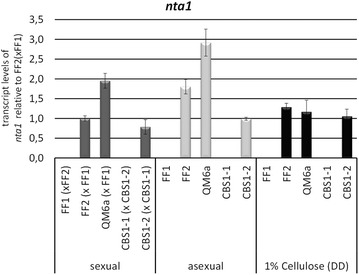
Regulation of *nta1* in both mating types. Strains were grown on malt extract agar for investigation of sexual development (contact stage, before fruiting body formation) and asexual development (strain alone on the plate). For liquid culture on cellulose (1% w/v), strains were grown in minimal medium in constant darkness for 72 h. Errorbars show standard deviations

Only one further gene showed mating type specific regulation, TR_121284, with more than 200-fold downregulation in MAT1-1 in both strain combinations. This gene is related to the *Saccharomyces cerevisiae* N-terminal amidase NTA1 (domain accession: cd07566; p-value 4.99e−141), which functions in the N-end rule protein degradation pathway. Hence, *T. reesei* NTA1 may be involved in mating type specific regulation of protein stability. RTqPCR confirmed the MAT1-2 specific regulation of *nta1* under conditions of sexual development, asexual development and growth on cellulose in liquid culture (Fig. 2). Since transcript levels of nta1 in MAT1-1 were under the detection level of our assay, the difference between MAT1-1 and MAT1-2 is at least 50000fold and signals for MAT1-1 in our transcriptome data can be considered background.

### Female sterility related gene expression under different conditions

**Fig. 3:**
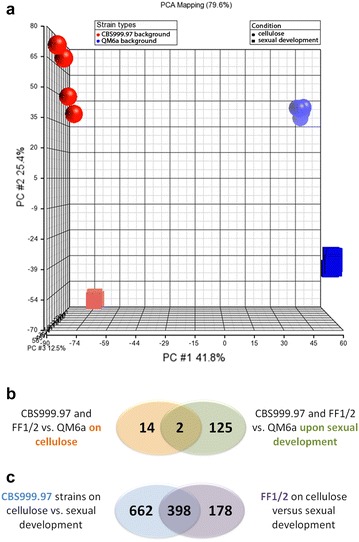
Comparision between cellulose and sexual development. **a** PCA analysis of gene expression patterns of different strains (CBS999.97 MAT1-1, CBS999.97 MAT1-2, FF1, FF2, QM6a) and upon growth on cellulose in liquid media as well as under conditions of sexual development. **b** Venn diagram of the comparison between genes differentially regulated between female fertile strains and QM6a versus the same gene set upon sexual development. **c** Venn diagram of the comparison between genes differentially regulated between cellulose and sexual development in CBS999.97 versus FF1/FF2 upon growth on cellulose compared to sexual development. Overlapping areas in the Venn diagrams show differentially regulated genes under both conditions or in both strains

**Fig. 4:**
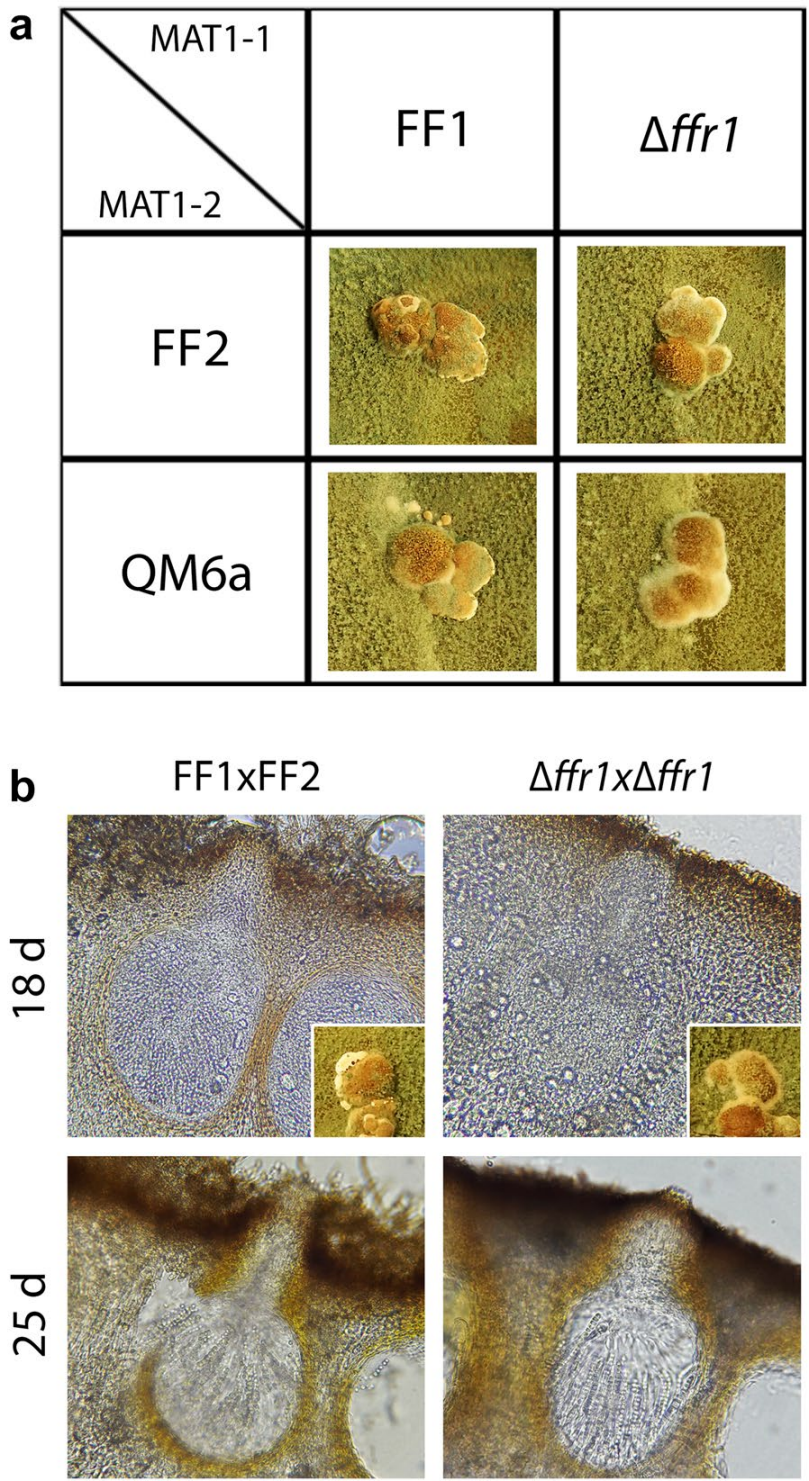
Relevance of FFR1 for sexual development. **a** Strains were crossed under standard conditions and showed normal sexual development. **b** If *ffr1* is lacking in both mating partners, fruiting body formation is delayed and fruiting bodies of this cross are not yet mature after 18 days. After 25 days, ascospore discharge commences as in the wildtype in the ∆*ffr1* crosses. Microscopic pictures show 400x magnification

In order to assess a more general defect in QM6a, we compared gene regulation in QM6a with that in the female fertile strains of both mating types under conditions of sexual development with differential regulation in the same strains (female fertile vs. QM6a), but upon growth on cellulose [10]. Principal component (PCA) analysis of these gene sets shows that QM6a patterns are closely related to those of FF1 and FF2 strains, which have largely the same background, and are distinct from CBS999.97, both with respect to growth on cellulose and under conditions of sexual development (Fig. 3a). 16 genes were differentially regulated between female fertile strains and QM6a on cellulose and 127 under conditions of sexual development (Fig. 3b). Only two genes showed significant regulation in this comparison, TR_35534 (roughly three-fold upregulation), a gene potentially involved in diterpene metabolism and TR_105242 (6- to 16-fold upregulation in female fertile strains), a putative SAM dependent methyltransferase. Therefore we designated TR_105242 as FFR1 (female fertility related 1) and deleted the encoding gene in FF1b in order to assess its function in sexual development (Fig. 4). ∆*ffr1* showed normal fruiting body formation and ascospore discharge in crosses with wild-type or QM6a (Fig. 4a). Upon crossing of ∆*ffr1* MAT1-1 with ∆*ffr1* MAT1-2, we found that fruiting body formation was delayed by 2 days and ascospore discharge by 3 days. After 18 days we found that the fruiting bodies of this cross were less mature than the wild-type, while after 25 days strains this difference was not visible anymore (Fig. 4b). Hence, although the absence of *ffr1* from the genome is relevant for sexual development, *ffr1* is neither essential for mating nor for female fertility.

### Gene regulation specific for sexual development

The availability of datasets of the same strains under conditions of sexual development and cellulase gene expression enabled us to narrow down the gene set specific for sexual development. Genes with highly regulated expression under sexual development conditions compared to cellulase conditions are expected to be more specific for sexual development. We compared gene regulation in CBS999.97 strains upon growth on cellulose versus under sexual development conditions and made the same comparison separately for FF1/FF2 strains to consider the different strain backgrounds and increase the significance of results (Additional file 3). 388 genes were more than fivefold (p-value threshold 0.01) differentially regulated under these conditions in CBS999.97 and FF1/FF2 strains (Fig. 3c). Of those, 168 were up-regulated on cellulose versus sexual development and 220 were down-regulated (Additional file 3).

No significant functional enrichment was detected in the gene set downregulated upon sexual development (up on cellulose, 168 genes), with the exeption of an enrichment in unclassified proteins (p-value 6.80e−09). However, this gene set contains 10 CAZyme encoding genes including *rgx1* (rhamnogalacturonase) and *xyn5* (xylanase; [30]), three genes involved in asexual development, the cellulose specific gene *ooc1* [31], the hydrophobin genes *hfb2*, *hfb3* and *hfb5*, the ceratoplatanin encoding gene *epl1*, involved in elicitation of plant responses [32], as well as the polyketide synthase encoding *pks4* gene (Additional file 3).

The gene set of upregulated genes upon sexual development (down on cellulose) is enriched in functions in metabolism (p-value 3.88e−07), particularly nitrogen, sulphur and selenium metabolism (p-value 3.34e−05), secondary metabolism (p-value 1.47e−13), C-compound and carbohydrate transport, amino acid transport and peptide transport (p-values below 3.5e−03), electron transport (p-value 8.61e−05) and fruit body development (p-value 4.15e−03) (Additional file 3). Twenty CAZyme encoding genes including several alpha- and beta-glycosidases and a chitinase were in this gene set as well as 8 PTH11 like G-protein coupled receptor encoding genes, 8 genes involved in secondary metabolism including several polyketide synthases, and 9 transcription factor genes. Interestingly, the whole SOR cluster with exception of the transcription factor gene *ypr2* [23, 33], which was recently found to be responsible for biosynthesis of the sorbicillin compounds trichodimerol and dihydrotrichotetronine [23], is upregulated on sexual development compared to growth on cellulose with fold regulations of around 20- up to 70-fold (Additional file 3).

Ten genes showed contrasting regulation between sexual development specific genes in CBS999.97 and strains with QM6a background. They include a candidate alpha xylosidase and *cel3d*, the alcohol oxidase gene *aox1* as well as two transporter genes (Additional file 3).

### Resource distribution specific to sexual development

**Fig. 5:**
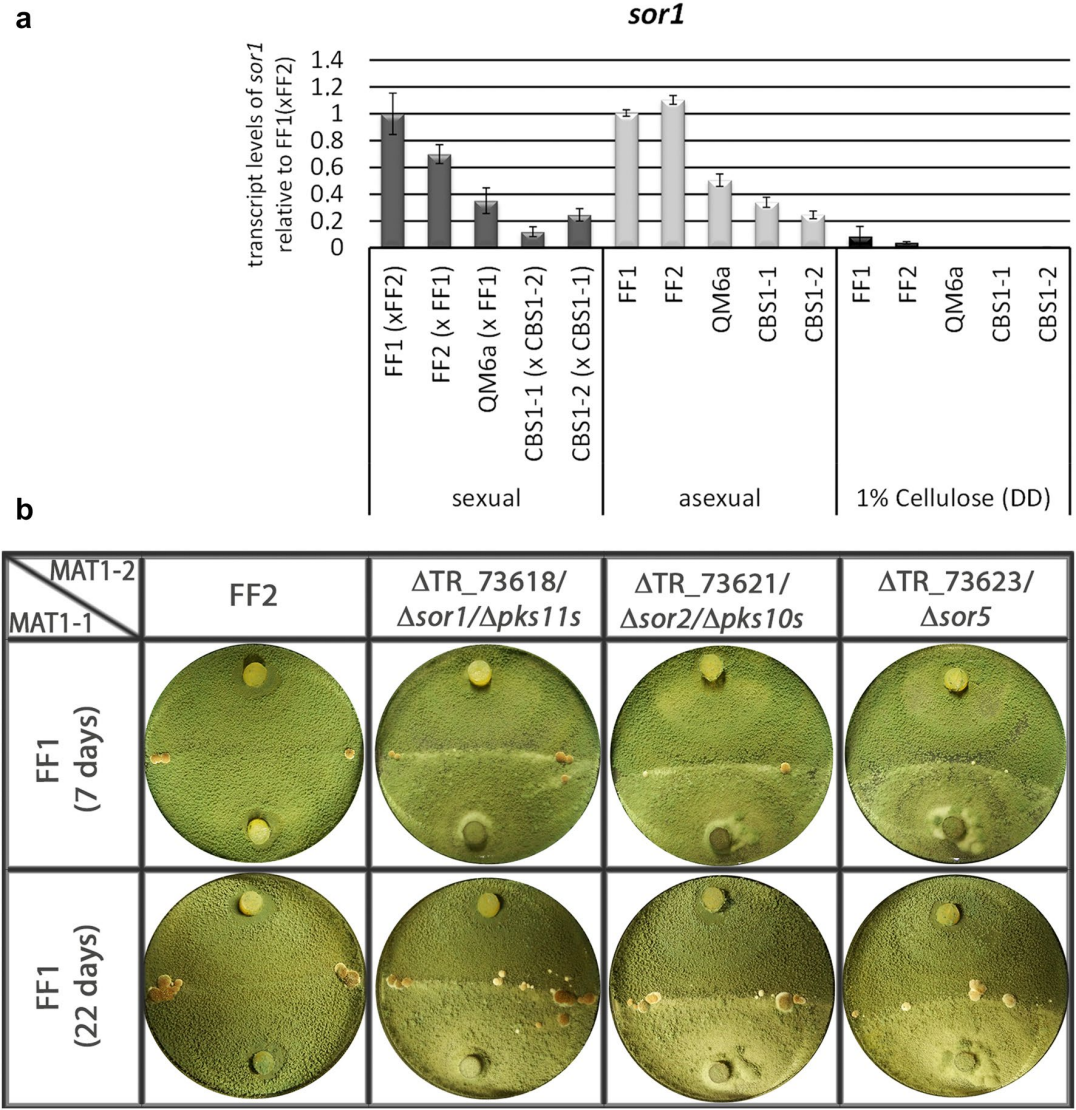
Relevance of SOR cluster genes for sexual development. **a** Transcript levels of *sor1* under conditions of sexual or asexual development or on cellulose. Strains were grown on malt extract agar for investigation of sexual development (contact stage, before fruiting body formation) and asexual development (strain alone on the plate). For liquid culture on cellulose (1% w/v), strains were grown in minimal medium in constant darkness for 72 h. Errorbars show standard deviations. **b** Analysis of sexual development in strains lacking biosynthetic genes of the SOR cluster. Strains grown in light cycles at 22 °C are shown after 7 or 22 days. Fruiting body formation started after 7 days, no changes were observed after 22 days. Ascospore formation was indistinguishable from wild-type

Increased transcript levels under a certain condition represent the first step to high level expression of the respective genes i.e. biosynthesis of the gene products. This investment of resources can be considered a preparation to exert the associated functions if translation and processing continues. However, a considerable number of highly expressed genes are unspecific housekeeping genes. Therefore we selected the 1000 genes with the highest transcript levels of CBS999.97 upon growth under conditions of sexual development and removed those that are among the 1000 most highly transcribed ones on cellulose. From this gene set we selected those genes that fulfilled the same criteria in FF1/2. Ninety four genes remained, which are likely to represent the most strongly expressed genes under sexual development conditions (Additional file 4). This gene set contains 5 CAZyme encoding genes, 2 PTH11-type G-protein coupled receptors, a protein phosphatase, 7 transcription factors and three transporters (Additional file 4). Among the transcription factor genes, a homologue of the *N. crassa* grainy head like transcription factor encoding *csp*-*2*, which is involved in conidial separation, development and cell wall remodeling [34], was found. However, the most interesting finding was the high level transcription of the three biosynthetic genes of the recently described SOR cluster [23, 33, 35] with TR_73618/*sor2/pks10s*, TR_73621/*sor1/pks11s* and TR73623/*sor5* being among the 10 genes with highest overall transcript levels in CBS999.97. We therefore tested transcript abundance of *sor1* under conditions of sexual development (contact stage, before fruiting body formation) compared to cellulose (Fig. 5a). The strong overexpression of *sor1* upon growth under conditions favouring sexual development compared to liquid culture on cellulose was confirmed. However, testing transcript abundance in the absence of a mating partner showed similarly high transcript levels (Fig. 5a). Hence, for *sor1* a specific significance for sensing of a mating partner is not supported, albeit a relevance for growth under conditions favouring sexual development cannot be excluded.

### Analysis of the function of the SOR cluster in sexual development

As the biosynthetic genes of the SOR cluster show particularly high transcript levels, we were interested in its relevance for sexual development. Also the corresponding up-regulation obtained for CBS999.97 and FF1/2 strains upon sexual development supports an importance for sexual development.

This cluster is responsible for light modulated production of the yellow compounds trichodimerol and dihydrotrichotetronin [23]. For regulators, similar expression levels as the regulated genes have been reported previously in *T. reesei* [20, 36] and were expected also in this case. Interestingly, the most important regulators of the cluster, *ypr1* and *ypr2* [33], are not among those with the highest transcript levels in CBS999.97, although their expression level upon sexual development is still higher than on cellulose. Additionally, differential regulation between CBS999.97 and FF1/FF2 occurs, which was not the case for the SOR biosynthetic genes. Therefore we consider it possible that other transcription factors contribute to regulation of the SOR cluster under conditions of sexual development. In order to identify candidates for such a function we performed a coregulation analysis. Coregulated genes are enriched in aromate metabolism (p-value 3.07e−03) and secondary metabolism (p-value 2.72e−04), supporting the hypothesis that secondary metabolism is highly important under sexual development conditions. We found several transcription factor encoding genes with a similar regulation pattern as TR_73618/*sor2/pks10s*, TR_73621/*sor1/pks11s* and TR73623/*sor5* with consistently high regulation in CBS999.97 and FF1/FF2 under sexual development versus low levels on cellulose. However, none of them reached comparably high transcript levels as these genes. Promising candidates for a contribution to regulation of *sor1*, *sor2* and *sor5* upon sexual development are TR_71823, TR_1941, TR_60761, TR_56141 and TR_3449.

Due to the high expression levels of *sor1*, *sor2* and *sor5*, we checked whether deletion of these genes, which abolishes or strongly reduces production of trichodimerol and dihydrotrichotetronin [23], would be essential for sexual development. Crossings with mutants in these genes showed that *sor1*, *sor2* and *sor5* are not essential for fruiting body formation and ascospore discharge of *T. reesei* (Fig. 5b). However, we observed that in the absence of *sor5* fruiting body formation is somewhat delayed (Fig. 5b). Hence neither these genes nor their biosynthetic products are essential for sexual development in *T. reesei*, but may have a beneficial influence.

## Discussion

Since the discovery of sexual development in *T. reesei*, the environmental conditions supporting this process as well as gene regulation required for mating to happen are subject of ongoing investigations [14]. Here we studied gene regulation patterns under conditions of sexual development in strains of different genetic background and compared them to the female sterile isolate QM6a. While a number of interesting targets for investigating female sterility of QM6a emerged from this study, the most striking finding was the differential regulation of genes involved in secondary metabolism in several of our evaluations. Hence, our analysis indicated an importance of secondary metabolism for sexual development both due to a strong enrichment in most highly expressed genes upon sexual development as well as consistent elevated transcript levels in a comparison of sexual development conditions with growth on cellulose.

Connections between development and secondary metabolism have been subject to intense research in fungi for decades [37–39] with the velvet family of proteins as important connecting factors [40]. Additionally, the *A. nidulans* transcription factor NsdD [41, 42] and its homologues, like SUB-1 in *N. crassa* [43], emerged as regulators of secondary metabolism and development [44–46].

In *T. reesei*, the light dependent transcription factor SUB1, a homologue of NsdD, was only recently shown to be required for female fertility and plays a role in regulation of the pheromone system and secondary metabolism [47]. SUB1 thereby influences abundance of a product of the SOR cluster, trichodimerol [23] in darkness, but not in light upon growth on cellulose [47]. In general, deletion of *sub1* causes altered abundance of secondary metabolites under different growth conditions, including sexual development albeit the nature of these metabolites remains to be determined in most cases [47].

LAE1 is a further known regulator of secondary metabolism and required for formation of the yellow pigment in *T. reesei* and at the same time essential for cellulase gene expression [48]. Therefore it can be assumed that LAE1 targets the SOR cluster as well. In *A. nidulans*, lack of laeA negatively influences sexual development, and the defect becomes even more severe if the *T. reesei* homologue is expressed in such a strain [49]. Deletion of *lae1* abolishes sporulation [48] and although a function in mating is likely, it is not yet known whether LAE1 influences sexual development in *T. reesei* as well.

Functions in both development and secondary metabolism have also been shown for VELVET, which controls the ratio of sexual/asexual development in response to light in *Aspergillus nidulans* [50]. However, VeA only has a small effect on the SUB1 homologue NsdD [50]. Also in *T. reesei*, the VeA homologue VEL1 has functions in sexual development as well as in secondary metabolism [22]. There, we could show a specific change in secondary metabolite patterns if a mating partner was present on the same plate. Consequently, VEL1 regulates chemical communication in *T. reesei* [22]. The nature of the secondary metabolites crucial for this communication is not yet known.

Our study revealed a considerable importance of genes involved in secondary metabolism during sexual development in *T. reesei*. Interestingly, the products of the SOR cluster, with the associated genes being highly expressed under conditions of sexual development in *T. reesei*, appear to be not essential for sexual development, because deletion of the biosynthetic genes shows that they are not required for mating (Fig. 5). Consequently, it is also unlikely that the sorbicillin derivatives biosynthesized by the SOR cluster enzymes are essential for development associated chemical communication.

The importance of the high level transcription of the SOR cluster genes upon sexual development can therefore be rather attributed to a protective mechanism, which can protect fruiting bodies against predators or competitors as suggested previously [51]. Our results are in agreement with earlier data showing that deletion of polyketide synthase genes does not abolish sexual development [52].

In our analysis, effects of the carbon source as well as the difference between cultivation on solid or in liquid media have to be kept in mind. However, available transcriptome data do not support a regulation of the SOR cluster genes here other than specific to development: *sor1*, *sor2* and *sor5* are up-regulated upon growth on cellulose and glucose compared to glycerol, lactose or sophorose [24]. Therefore the specific up-regulation under mating conditions cannot be attributed simply to an altered carbon source, because expression levels on carbon sources other than cellulose are similar or lower than on cellulose [24], but not strongly elevated as the results for sexual development shows.

Upon growth on cellulose, the SOR cluster genes are down-regulated upon growth in light compared to darkness [23, 53]. In contrast, in our study, these genes are strongly up-regulated under conditions of sexual development (light, malt extract medium) compared to growth on cellulose (darkness). We conclude that indeed conditions of sexual development, but not merely illumination or a specific carbon source are responsible for the elevated transcript levels of *sor1*, *sor2* and *sor5*.

## Conclusions

In summary we found that generally, the strain background (CBS999.97 versus QM6a) is more relevant for gene regulation than the mating type. While in different strain backgrounds a number of genes are regulated according to mating type, consistent regulation in the different strains and hence in general in T. reesei appears limited to the pheromone system and mating type genes. Our data support the role of secondary metabolism for chemical communication as postulated earlier. Hence the interrelationship between secondary metabolism and sexual development warrants further investigation.

## Methods

### Strains and cultivation conditions

QM6a (ATCC13631; [2]), CBS999.97 MAT1-1, CBS999.97 MAT1-2 [6], FF1 and FF2 [22, 24] along with sister strains from different crossing lines of FF1 and FF2, (FF1a, FF1b, FF2a, FF2b) were used in this study. FF1 and FF2 were prepared by backcrossing the female fertile CBS999.97 MAT1-1 with female sterile QM6a 10 times in order to acquire sexual competence while retaining the QM6a phenotype [22]. For testing the influence of SOR1, SOR2 and SOR5 on sexual development, the respective deletion strains [23] were used (Table 1). Strains were propagated on malt extract medium. For inoculum preparation, strains were grown in constant darkness for 10 days, thereby avoiding an influence of random light pulses or circadian rhythmicity on gene regulation. Crossings were done on malt extract medium (2% w/v) in light–dark cycles at 22 °C as described previously [6].

**Table 1:** Strains used in this study

Strain	Code	Characteristics	Source/reference
CBS999.97 MAT1-1	CBS1-1	Wild-type MAT1-1, female fertile	[6]
CBS999.97 MAT1-2	CBS1-2	Wild-type MAT1-2, female fertile	[6]
FF1a, FF1b	FF1	Backcrossed wild-type strain MAT1-1	[22]
FF2a, FF1b	FF2	Backcrossed wild-type strain MAT1-2	[22]
QM6a		Wildt-type MAT1-2, female sterile	[2]
QM6a ∆*sor1*		∆*sor1*∆ku80::hph + MAT1-2	[23]
QM6a ∆*sor2*		∆*sor2*∆ku80::hph + MAT1-2	[23]
QM6a ∆*sor5*		∆*sor5*∆ku80::hph + MAT1-2	[23]
FF1 ∆*ffr1*		∆*ffr1*∆ku80::hph + MAT1-1	This study
FF2 ∆*ffr1*		∆*ffr1*∆ku80::hph + MAT1-2	This study

For transcriptome analysis, strains were grown under conditions facilitating sexual development as described previously [6, 22]. Strains with similar genetic background were combined in crosses (CBS999.97 MAT1-1 × CBS999.97 MAT1-2; QM6a MAT1-2 × QM10a MAT1-1 [6]; FF1a × FF2a; FF1b × FF2b). 40 plates per combination were used, strains were harvested separately at subjective noon at the contact stage, separated in two groups per combination and pooled resulting in two biological replicates based on 20 individual plates each. Contamination of samples by the other strain of different mating type on the same plate was tested as described previously [15] and was generally below 0.1%.

### Transcriptome analysis

Isolation of total RNA and quality control using the Bio-Rad Experion system (Hercules, CA, USA) was done as described previously [54]. The quality threshold for use of samples in transcriptome analysis was set to a RIN (RNA integrity number) value of 9. The NimbleGen (Madison, WI, USA) gene expression full service was used as described previously [10, 21] using custom arrays for QM6a and CBS999.97. Data are available at NCBI GEO (https://www.ncbi.nlm.nih.gov/geo/) under accession number GSE89104.

Bioinformatic analysis was performed using the Partek Genomics Suite 6.5 (Partek Inc., St. Louis, MO, USA), which applies ANOVA analysis for identification of statistically significant gene regulation. Datasets were grouped according to the specific scientific question and treated as replicates (for example, all strains with the CBS999.97 background were compared to all strains with the QM6a background).

Hierarchical clustering analysis and analysis of expression patterns was performed using the open source software HCE3.5 [55]. The online analysis platform at MIPS (http://mips.helmholtz-muenchen.de/funcatDB/) [56] was used for functional category analysis of gene sets with its latest version of May 2014. The p-values shown with this analysis indicate the extent of significant enrichment of a given gene group within a gene set of regulated genes.

Annotation of genes listed in additional files was done using the comparative genome study on *T. reesei*, *T. atroviride* and *T. virens* [3] and complemented by data provided in [57].

### Quantitative reverse transcription PCR

RTqPCR was performed as described previously [23, 54] using *rpl6e* and *sar1* as reference genes, which were shown to be appropriate for the conditions we used here [47]. For analysis of *sor1* primers RT_73621_F (5′ GCAACCTCGTCGATTTGGCTGC 3′) and RT_73621_R (5′ AAGTGTCTCGAGAAGGACGCGC 3′) [23] and for *nta1*, primers 121284RTq1F (5′ ACTCTCATGCTGAATGTTCAC 3′) and 121284RTq1R (5′ TGGAGGCAGAGTAGCTCAC 3′) were used. Data were evaluated using the CFX Maestro software (Bio-Rad, Hercules, USA).

### Gene deletion

The gene encoding TR_105242, *ffr1*, was deleted in the female fertile strain FF1b. Therefore, the vector pDEL105242 was constructed by yeast recombination cloning as described in [58] for selection using hygromycin. Transformation and selection of deletion strains was performed by protoplasting as described previously [59]. The 5′ flanking region was amplified using primers 105242_5F (5′ GTAACGCCAGGGTTTTCCCAGTCACGACGGCGTAGGCTACTCAGTCTGC 3′) and 105242_5R (5′ ATCCACTTAACGTTACTGAAATCTCCAACATCCTGTGTCACTCCTATCC 3′) and the 3′ flanking region was amplified using primers 105242_3F (5′ CTCCTTCAATATCATCTTCTGTCTCCGACATATGGAGGTCGAGGAAACC 3′) and 105242_3R (5′ GCGGATAACAATTTCACACAGGAAACAGCCTCCGAGTTGCAATAGTAGC 3′). Removal of the open reading frame was confirmed by PCR using primers 105242_qF (5′ ATTCGCACGACCACTCTCAC 3′) and 105242_qR (5′ CGCCATGCTTGGAGATTGTG 3′).

## Additional files

**Additional file 1.** Comparative analysis between genes regulated in female fertile and female sterile strains.

**Additional file 2.** Genes differentially regulated between mating types.

**Additional file 3.** Comparative analysis of gene regulation between growth on cellulose and under conditons of sexual development.

**Additional file 4.** Genes with highest transcript levels under sexual development conditions.
